# Docosahexaenoic and Eicosapentaenoic Intervention Modifies Plasma and Erythrocyte Omega-3 Fatty Acid Profiles But Not the Clinical Course of Children With Autism Spectrum Disorder: A Randomized Control Trial

**DOI:** 10.3389/fnut.2022.790250

**Published:** 2022-03-29

**Authors:** Maria Jose de la Torre-Aguilar, Antonio Gomez-Fernandez, Katherine Flores-Rojas, Pilar Martin-Borreguero, María Dolores Mesa, Juan Luis Perez-Navero, Mónica Olivares, Angel Gil, Mercedes Gil-Campos

**Affiliations:** ^1^Pediatric Research and Metabolism Unit, Reina Sofia University Hospital, Maimónides Institute for Biomedical Research of Córdoba (IMIBIC), University of Córdoba, Córdoba, Spain; ^2^Department of Child and Adolescent Clinical Psychiatry and Psychology, Reina Sofia University Hospital, Maimónides Institute for Biomedical Research of Córdoba (IMIBIC), Córdoba, Spain; ^3^Department of Biochemistry and Molecular Biology II University of Granada, University of Granada, Granada, Spain; ^4^Instituto de Investigación Biosanitaria IBS.GRANADA, Complejo Hospitalario Universitario de Granada, Granada, Spain; ^5^Biomedical Research Center, Institute of Nutrition and Food Technology “José Mataix,” University of Granada, Parque Tecnológico de la Salud, Granada, Spain; ^6^CIBERER (Ciber Rare Diseases), Instituto de Salud Carlos III (ISCIII), Madrid, Spain; ^7^Biosearch Life, Granada, Spain; ^8^CIBEROBN (Physiopathology of Obesity and Nutrition), Instituto de Salud Carlos III (ISCIII), Madrid, Spain

**Keywords:** autism spectrum disorder, diet, food and nutrition, docosahexaenoic acid, fatty acids, cytokines

## Abstract

**Background:**

The pathogenesis of autism spectrum disorder (ASD) is under investigation and one of the main alterations relates to the metabolic and inflammatory system dysfunctions. Indeed, based on a possible deficit of omega-3 fatty acids (FAs) of patients with ASD and looking for an anti-inflammatory effect, dietary supplements with omega-3 fatty acids have been proposed. We aimed to evaluate differences in plasma and erythrocyte FA profiles and plasma cytokines in patients with infantile ASD after supplementation with docosahexaenoic (DHA) and eicosapentaenoic (EPA) acids or placebo and both compared at baseline with a reference healthy group.

**Methods:**

A double-blind, randomized placebo-controlled intervention with DHA/EPA for 6 months was carried out in 54 children between 2 and 6 years diagnosed with ASD. They were selected and randomly assigned into two groups: 19 children received 800 mg/day of DHA and 25 mg/day of EPA, or placebo. In addition, another reference group of 59 healthy children of the same age was included. Plasma lipids and cytokines, and FA profiles in plasma and erythrocytes were measured at baseline and after 6 months of treatment in ASD children, and at baseline in the reference group.

**Results:**

There were no differences in demographic, anthropometric characteristics, and omega-3 intake between the healthy reference group and the ASD children at baseline. Children with ASD showed the higher plasma percentages of palmitic acid and total saturated FA and lower total omega-6 polyunsaturated FA (PUFA) compared with healthy children. An increased level of DHA and reduced EPA level in erythrocytes were detected in the ASD group vs. the reference group. After 6 months of treatment, the ASD group that received DHA enriched product significantly increased the plasma and erythrocyte percentages of DHA, but no differences were observed in the clinical test scores and other parameters as plasma cytokines between the two groups of ASD related to the intervention.

**Conclusion:**

Spanish children with ASD exhibit an appropriate omega-3 FA status in plasma and erythrocytes. Neither a clinical improvement of ASD children nor a better anti-inflammatory or fatty acid state has been found after an intervention with DHA/EPA for 6 months. So, the prescription of n-3 LC-PUFA and other dietary supplements in ASD should be only indicated after a confirmed alteration of FA metabolism or omega-3 LC-PUFA deficiency evaluated by specific erythrocyte FA.

**Clinical Trial Registration:**

[www.ClinicalTrials.gov], identifier [NCT03620097].

## Introduction

Autism spectrum disorder (ASD) is a neurodevelopmental disorder characterized by alterations in communication and social interaction and by the presence of repetitive and restricted patterns of behaviors, activities, and interests (1). Although there are approved drugs for treating the comorbidities of ASD, there is no curative treatment (2). Some evidence supports that dietary supplements, such as antioxidants, vitamins, or omega-3 fatty acids (FAs) can promote cognitive development; however, the efficacy in improving ASD evolution is not sufficiently researched and documented (3).

Omega-3 long-chain polyunsaturated fatty acids (LC-PUFA), especially docosahexaenoic acid (22:6 n-3) (DHA), are the structural components of cell membranes, especially in the central nervous system. They contribute to neuronal growth and differentiation, synapses, visual acuity, or even the regulation of gene expression (4, 5). In addition, they regulate anti-inflammatory and oxidative stress systems (6). Docosanoids derived from DHA, namely, D-resolvins, protectins, and maresins are important lipid mediators in the resolution of inflammation (7) and particularly neuroprotection D1 inhibits neuronal apoptosis (8). Eicosapentanoic acid (20:5 n-3) (EPA), although present in the cell membranes of neural cells in lower amounts than DHA, has important functional roles as a source of anti-inflammatory molecules, namely, PGE3, LTB5, and E-resolvins (9).

Nowadays, there is a great interest in omega-3 FA supplementation in some neuropsychiatric disorders in adults but also others that debut during childhood or adolescence as attention deficit hyperactivity disorder (ADHD), anorexia nervosa, and ASD (10). Factors, such as the absence of curative treatment, or the high cost of early care therapies, together with the difficult interpretation of its long-term success, contribute greatly to the families seeking alternatives to traditional medicine as a solution for their children. Usually, omega-3 FA treatment, associated with others multivitamin supplements, gluten-free casein-free diet, or methyl B-12 injections, is widely used among patients with ASD (11–14). About the efficacy and safety of omega-3 FA in ASD, few studies with small samples of children, with different origins and doses of omega-3 FA, have reported contradictory results (15–19).

A possible alteration in the absorption and metabolism processes of PUFA has been hypothesized in ASD children to explain some symptoms (20, 21). Although three meta-analyses have evaluated the effects of omega-3 FA supplementations in ASD, the assessment of their efficacy is difficult for many reasons: variation in formulations, time of administration, and tests used to measure efficacy. In fact, two of them could not show any benefit in administering omega-3 (6, 22). However, Cheng et al. concluded that supplementation with omega-3 FA may improve hyperactivity, lethargy, and stereotypy in patients with ASD, although it does not seem to ameliorate overall functioning (23). Omega-3 FA supplements vary in the dose and source of the FA, especially in DHA and EPA, doses ranging from 0.2 g/day DHA to 1.5 g/day EPA + DHA and time courses from 6 weeks to 6 months. The sources of omega-3 FA supplements included fish oil and algal oil (23). Based on the current evidence-based treatment guidelines, a combination of EPA + DHA with 1.3–1.5 g/day for 16–24 weeks has been recommended to treat ASD (24). Indeed, a great interest is emerging around the DHA supplementation in ASD, based on a possible deficiency in the intake explained by restrictions associated with the eating behavior of these patients, especially at early ages (25), or in their absorption and metabolism, e.g., potential FA tissue desaturases’ alterations (26).

Another important reason to continue in this research field is the limited studies correlating n-3 LC-PUFA supplementation and its potential anti-inflammatory activity. The benefit obtained with n-3 LC-PUFA supplementation in ASD children is considered to be through its role in modulating inflammation (27). Some studies relate symptom severity, plasma cytokine levels, and FA profiles in ASD children (28). However, few studies correlate FA supplementation, cytokine profile, and pre- and post-intervention clinical assessment with FA.

Based on the above, there would be lower fatty acids at baseline and/or defective enrichment in EPA/DHA after following a supplementation. Hence, the present clinical trial aimed to evaluate plasma and erythrocytes FA profiles before and after supplementation with DHA in pediatric patients with ASD, according to recent recommendations for dosage and treatment time and both compared at baseline with a reference healthy group for dietary habits and fatty acid status to explore whether an alteration in PUFA profiles could be present leading to the quantitative or functional deficits, which could result in a derangement of the disease clinical course. In addition, we also assessed whether this supplementation with DHA + EPA leads to the changes in the plasma inflammatory cytokine profile as the underlying mechanism of its effect, and qualitatively assessed the clinical improvement with validated clinical tests.

## Materials and Methods

### Study Design

This is a parallel, double-blind, randomized, placebo-controlled trial in patients between 2 and 6 years old diagnosed with ASD by the Child and Youth Mental Health Unit of Córdoba, Spain, based on the DSM-5 criteria and the observation scale for the autism diagnosis [Autism Diagnostic Observation Schedule (ADOS)] (1, 29). It was a phase III, non-inferiority, single-center national study with an independent character without commercial interest.

**FIGURE 1:**
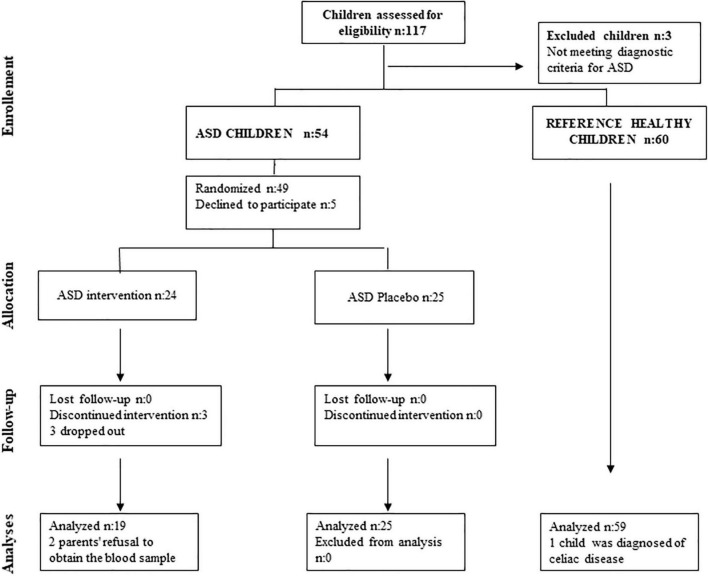
Flow chart for participants and treatment arm allocation according to CONSORT norms.

Patients with ASD were included in the trial consecutively and were divided into two parallel groups according to the randomization generated by the SIGESMU^®^ computer program with allocation randomized 1:1. Thus, in a double-blind system, one subgroup received an omega’-3 supplement of 800 mg of DHA and 25 mg of EPA per day and the other received a placebo with similar lipid characteristics except for DHA and EPA content, for 6 months. The composition for both formulas is included in Table 1 and details are explained below in the Study Intervention Section. A clinical evaluation and analytical study were performed at baseline and after 6 months at the end of the intervention. In addition, a controlled prospective observational case-control study was conducted comparing the plasma levels of the biochemical parameters between ASD children and a healthy reference group of children at baseline. The flowchart for participants and treatment arm allocation according to CONSORT norms is in Figure 1.

**TABLE 1:** Fatty acid composition of placebo and intervention oils.

Fatty acid	Placebo%	Intervention oil%
14:0	0.04	3.95
15:0		0.93
16:0	3.62	18.96
16:1		4.55
17:0		1.05
18:0	3.92	4.84
18:1 n-7		2.34
18:1 n-9	80.91	14.51
18:2 n-6	9.74	2.00
18:3 n-3	0.12	0.88
18:4 n-3		0.95
20:0	0.32	0.35
20:1 n-9	0.22	1.36
20:4 n-6		2.39
20:4 n-3		0.49
20:5 n-3		8.10
22:0	0.81	0.11
22:1 n-11		0.63
22:4 n-6		1.48
22:5 n-3		1.49
22:6 n-3		25.84
24:0	0.30	
Others		1.8

*Results are expressed in percentages. Data are derived from gas-liquid chromatography coupled to mass spectrometry.*

### Participants

Pediatrics patients (2–6 years) diagnosed with ASD were considered for the recruitment. The reference group of healthy children of the same age, since in many of the study variables, there are no reference values in childhood, was selected from among those who attended a hospital consultation to rule out some minor pathology that required laboratory tests, with the confirmed absence of disease. Participants were recruited for the study from January to December 2015.

The inclusion criteria were children between 2 and 6 years of age with a diagnosis for ASD according to the DSM-V criteria and the ADOS in the ASD group, and health confirmed status in the reference group, with the informed consent signed by one of the parents or legal representatives.

Exclusion criteria: Children under 2 years old; children diagnosed with ASD over 6 years old; the coexistence of another diagnosis associated with autism; patients who were receiving some type of supplement or concomitant medication that did not allow a washout period or patients diagnosed with other pathologies or with medication that might affect the study variables (inflammation or lipid profile).

The withdrawal criteria were children who did not maintain adequate adherence or compliance to the treatment as assessed by the researchers or that could not complete the biochemical analysis or the appearance of neurological pathology or unable to despite a single diagnosis of ASD.

### Study Intervention

The nutritional supplement, EUPOLY-3^®^ DHA Infant, was a product marketed following the regulations of the European Union (EU). This supplement was provided free of charge by Biosearch SA (Granada, Spain), as well as the placebo, in identical containers labeled with a numerical code and a product code: EUP007. Placebo oil was based on sunflower oil and intervention oil on fish oil. Both oils were stabilized with a 0.1% mixture of natural tocopherols, 1% soy lecithin, and 0.6% ascorbyl palmitate, and contained a colored lemon flavor in sufficient quantity to match the aroma and color of both products. The containers of both placebo and intervention products were packaged in indistinguishable small bottles and coded so that they were blinded to both participants and health personnel involved in the intervention. The levels of toxins and heavy metals in the raw material were controlled and below the EU limits. The supplement and the placebo kept the rest of the FA profile similar (e.g., content of the essential FA linoleic and linolenic acids). For the present trial, a suitable formulation was designed especially for preschool children with autism in a liquid form and a concentrated dose to ingest orally in the smallest amount, the 800 mg of DHA or placebo doses per day. The intake was recommended during the main meal once per day. The adherence plan for the intervention included telephone calls from the pediatricians to the tutors every 2 months to assess evolution, incidents, and evaluation of the administration and tolerance of the product.

### Biases

The measures to try to eliminate the bias factors were: first, the random assignment of patients to the different study treatments, allocation ratio 1:1 using the SIGESMU^®^ computer program. The design was double-blind, so neither the families nor the researchers knew the composition of the product used. To ensure that patients and researchers remained blind, the preparation of the placebo was carried out by the company that prepared the product, with the same packaging and labeling, and its organoleptic characteristics as an active drug. To avoid potential biases due to patient withdrawal after randomization, an intention-to-treat analysis was performed. Other actions to avoid bias were: homogenize criteria and diagnose patients with ASD with the greatest rigor to avoid any association with other cognitive alterations that could alter the results or affect non-compliance with treatment and to avoid variations in the conditions of the trial once it has begun.

### Ethical Aspects

This study was approved by the Ethics Committee of the Reina Sofia University Hospital of Cordoba, Spain, and follows the rules of Law 14/2007 on Biomedical Research and the Organic Law 15/1999, RD 1720/2007 on the protection of personal data as well as international rules for research using samples from human beings. The parents or legal guardians of the participants accepted their inclusion in the study, signing the approved protocol. The confidentiality of the data obtained and any personal data used in this study have been kept and respected.

### Clinical Evaluation

To evaluate the dietary pattern of ASD and reference children, a previously modified, adapted, and validated food frequency questionnaire (FFQ) with the portion sizes and food groups usually consumed by the Spanish child population was used (30). The adequacy of food consumption was evaluated using the Nutritional Objectives of the Consensus Document of the Spanish Community Nutrition Society; these questionnaires included one item about the dietary type and amount of supplementation (31). The intake of energy and nutrients was estimated from the data obtained through three non-consecutive 24-h dietary registrations (24-h-DR), registered by parents, such as 2 weekdays and 1 weekend day, following the Guidance on the EU Menu Methodology of the European Food Safety Agency (EFSA) (32).

Parents/guardians directly provided the answers to questionnaires and 24 h registrations (33). Physical examination with special emphasis on neurological and nutritional status and clinical evolution was carried out. Anthropometric measurements (weight, height, and body mass index) were obtained using standard techniques. All these measures and the biochemical analysis were performed at baseline and at the end of the intervention in the ASD group, and only at baseline time in the healthy reference group.

All the ASD children underwent an initial developmental clinical interview, which identified the core symptomatology of ASD according to DSM-5 clinical diagnostic criteria. Additionally, the following tests were administered to all children at baseline and at the end of the trial: (a) Autism Diagnostic Observation Schedule (ADOS). (b) Pervasive Developmental Disorders Behavior Inventory (PDDBI) (34). (c) Childhood Autism Rating Scale Test (CARS-2) (35). (d) Strengths and Difficulties Questionnaire (SDQ) (36).

### Biochemical Analysis

After overnight fasting, blood samples were collected from autistic children and healthy controls from the antecubital vein into 6-ml blood collection tubes containing ethylenediaminetetraacetic acid (EDTA) and processed within 2 h. After centrifugation at 3,500 g for 10 min, plasma was divided into aliquots from sampling and then frozen at −80°C until the analysis. The cell pellet was immediately washed three times with a 0.9% NaCl isotonic solution, and the packed erythrocytes were collected and stored at −80°C until lipid extraction. Blood count and general biochemical were performed to confirm the absence of other diseases. C-reactive protein (C-CRP) was measured as an inflammatory marker. These analyses were performed in the hospital laboratory using colorimetric, enzymatic, kinetic, indirect potentiometry or immunoturbidimetric methods previously standardized, using an automatic autoanalyzer (Roche-Hitachi Modular PYD autoanalyzer, Roch Laboratory Systems, Mannheim, Germany. The plasma lipid profile includes triacylglycerols (TG) (CV: 1.5%), cholesterol (c) (CV: 0.9%), high-density lipoprotein cholesterol (HDL-c) (CV: 0.8%), and low-density lipoprotein cholesterol (LDL-c) (CV: 1.5%), that were measured using an automatic analyzer (Accelerator APS system. Architect-c16000 Abbott Laboratories, S.A., Illinois, United States).

### Plasma and Erythrocyte Fatty Acid Analysis

Lipids from 0.2 ml plasma were extracted and fatty acid transmethylated in a single step according to the Lepage and Roy methodology (37). Briefly, a direct methylation procedure was carried out in 5.0 ml of methanol-acetyl chloride 50:1 (v/v). To stop the reaction 3 ml of 6.0% K_2_CO_3_ was added. After adding 150 μl of hexane, shaking and centrifugation, the upper phase was separated and dried under nitrogen.

For the extraction of erythrocyte lipids, about 1 ml of washed cells were successively treated with 3 ml of isopropanol containing 50 mg/L butyl-hydroxytoluene as an antioxidant, 2 ml of isopropanol and 2 ml of hexane. After centrifugation for 10 min at 3,000 g at 4°C, the upper phase of hexane was collected and the infranatant was re-extracted three times with 2 ml hexane. The hexane extracts were combined and dried under nitrogen. For the methylation of erythrocytes fatty acids, we followed a similar process to that described above for plasma (37).

The methylated fatty acids were resuspended in 100 μl of hexane and 1 μl injected into a Hewlett Packard HP5890 Series II chromatograph (Hewlett Packard, Palo Alto, CA, United States), with a capillary column (60 m × 32 mm inner diameter; 20 μm film thickness) impregnated with SP2330 FS (Supelco, Bellefonte, CA, United States). Running conditions were as described elsewhere (38). Fatty acid methyl esters were identified by the comparison of retention times with those of authentic standards run previously. Peaks were identified by comparison with known standards (Sigma, St Louis, MO) and the results.

Based on previous observations in the literature (39), relevant molecules previously described as associated with inflammatory processes and related to autism were selected for their analysis in plasma: interleukins (IL) IL-1beta, IL-6, and IL-8, monocyte chemoattractant protein-1 (MCP-1), tumor necrosis factor-alpha (TNF-α), myeloperoxidase (MPO), regulated on activation, normal T cell expressed and secreted (RANTES) [also known as chemokine (C-C motif) ligand 5, CCL5], cathepsin D, brain derived neurotrophic factor (BDNF), hepatocyte growth factor (HGF); nerve growth factor (NGF), plasminogen activator factor (PAI), and cell adhesion molecules [neuronal cell adhesion molecule (NCAM), intercellular adhesion molecule (ICAM), vascular cell adhesion molecule (VCAM)]. These biomarkers were analyzed by the simultaneous detection of multi-analytes using LINCOplex assay kits and Luminex xMAP Detection Technology following the manufacturer instructions (Linco Research, Inc., St. Charles, MO, United States).

### Statistical Analyses

To calculate the sample size, the GRANMO (2012) software was used considering the range in the plasma levels of DHA 0.97 ± 0.39%. We accepted an alpha type I error of 0.05 and a beta type II error of 0.2 (80% power) in a bilateral contrast to detect a difference equal to or greater than 0.3% units with an estimate of a loss to follow-up rate of 5%:

- For independent groups (healthy vs. ASD), 54 subjects were required in each group. The common standard deviation (SD) was assumed to be 0.5.
- For related groups (ASD with/without DHA intervention, and/or baseline vs. 6 months of treatment), 24 subjects were required in each group. Thus, 108 children in total, 54 healthy children and 54 children with ASD would be required to detect these differences.

Data were expressed as mean with SD [95% confidence intervals (*CI*s)], median with interquartile range (IQR), and categorical data as frequencies. The Kolmogorov–Smirnov goodness-of-fit test was used to test the distribution of each dataset for normality before analysis. The homogeneity of variance was estimated using Levene’s test and non-normally distributed data were transformed into approximate normal distributions by logarithmic transformations.

Demographics and clinical characteristics of participants and baseline FA levels were compared among groups (ASD vs. reference) using the Student’s *t*-test for unpaired samples for parametric data and by the Mann–Whitney *U*-test for data with the asymmetric distribution. Categorical data were analyzed by χ^2^ or Fisher’s exact test. “The analysis comparing the effects of treatment in FA levels and symptoms score for ASD over 6 months was conducted using ANOVA mixed-designs plus the Bonferroni correction for repeated measures.” Treatments and time were included as fixed effects and the interactions between interventions and time were also tested. If significant main effects or interaction effects were observed, *post hoc* analysis with Sidak correction was performed. Associations between the severity of ASD symptoms and nutritional status (considering FA) were assessed using the Pearson coefficient. The primary method of analysis is intention-to-treat. All statistical tests were two-tailed, and the values of *p* < 0.05 were considered to indicate statistical significance. Statistical Package SPSS, version 23 by IBM (SPSS Inc., Chicago, Illinois, United States), was used for these analyses.

## Results

### Patients’ Demographic and Anthropometric Characteristic

Initially, 57 ASD children were selected for this study. In the reference group, 60 children were initially recruited, and only one was excluded after a celiac disease diagnosis was performed (Figure 1). In the ASD subgroup with dietary intervention with DHA, 19 children were finally studied (3 did not continue with DHA supplementation and another 2 could not complete the analysis). Children who discontinued the DHA intervention were not included in the analysis. The placebo subgroup included 25 ASD children, and the reference group 59 (Figure 1).

Table 2 shows the demographic and anthropometric characteristics and the plasma lipid profiles of the healthy reference group and the placebo and intervention groups for ASD children at baseline. No differences were observed between ASD and the healthy reference children for any of these considered variables except for a decrease in serum triacylglycerol levels in the intervention group (60.5 ± 19.5 vs. 52.1 ± 12.8 mg/dl, *p* = 0.02).

**TABLE 2:** Demographic, anthropometric, and serum lipid profile in children with autism spectrum disorders (ASDs) compared with a control group and by subgroups by DHA intervention or placebo.

			ASD placebo n:25		ASD intervention *	
	Reference n:59	ASD n:54	Baseline	Final intervention	Baseline	Final intervention
Ages (Month)	48.8 ± 18.3	43.76 ± 11.2	41.9 ± 11.1		45.7 ± 11.2	
Sex (Boy)	46 (77%)	45(83%)	22 (88%)		14 (74%)	
Weight (kg)	17.1 ± 4.5	16.9 ± 3.5	16.9 ± 3.5		17.1 ± 3.6	
Height (cm)	101.9 ± 11.1	102.7 ± 8.3	101.8 ± 8.5		103.8 ± 8.3	
BMI (kg/m^2^)	16.2 ± 1.7	15.9 ± 1.7	16.1 ± 1.5		15.4 ± 1.8	
Cholesterol (mg/dl)	158.7 ± 31.6	168.2 ± 29.9	176.2 ± 29.3	173.2 ± 35.3	160.7 ± 29.6	166.1 ± 25.5
HDL cholesterol (mg/dl)	46.9 ± 12.9	47.1 ± 10.3	46.39 ± 11.2	48.3 ± 1	45.6 ± 8.8	49.2 ± 10.2
LDL cholesterol (mg/dl)	97.8 ± 24.8	107.3 ± 26.9	114.7 ± 25.8	111.6 ± 3	102.7 ± 28.5	106.1 ± 25.1
Triacylglycerol (mg/dl)	67.7 ± 31.7	67.3 ± 23.1	73.3 ± 26.7	64.4 ± 16.2	60.5 ± 19.5	52.1 ± 12.8^a^

*ASDs, autism spectrum disorders; BMI, body mass index; DHA, docosahexaenoic acid.*

*^a^p < 0.05 value intragroup. Data are given as the mean ± SD.*

*ASD intervention: 3 did not continue with DHA supplementation and another 2 could not complete the analysis. N baseline: 25; N final intervention: 19.

Dietary intake showed no significant differences between the intake of fish and shellfish that represent the most important food group contributing to omega-3 PUFA consumption: ASD group: 2.5 ± 1 servings per week vs. Reference group: 2.6 ± 1.1; *p* = 0.22. The average daily intake of EPA + DHA in ASD children was about 0.125 g/day, like that of the healthy population of reference.

### Plasma and Erythrocyte Fatty Acid Profiles Baseline and After Intervention

Tables 3, 4 show the FA profiles in plasma and erythrocytes, respectively. Data between the reference group and ASD children at baseline, and after 6 months of intervention between the ASD subgroups (DHA vs. placebo) were compared.

**TABLE 3:** Comparison of plasma fatty acid profile between reference group and ASD children.

Fatty acids (%)			ASD placebo n:25		ASD intervention n:19		*P*-value		
	Reference N:59	ASD total N:54	Baseline	Final intervention	Baseline	Final intervention	Intervention	Time	Time x intervention
**SFA**	**34.17 ± 5.62**	35.20 ± 3.72*	34.59 ± 2.71	34.06 ± 2.76	35.49 ± 4.58	34.65 ± 10.51			
Lauric acid 12:0	0.08 ± 0.17	0.05 ± 0.05	0.04 ± 0.05	0.05 ± 0.07	0.05 ± 0.05	0.07 ± 0.08			
Myristic acid 14:0	0.65 ± 0.28	0.62 ± 0.2	0.65 ± 0.22	0.61 ± 0.19	0.6 ± 0.19	0.58 ± 0.21			
Pentadecanoic acid 15:0	**0.09 ± 0.10**	0.14 ± 0.08*	0.14 ± 0.08	0.13 ± 0.09	0.14 ± 0.08	0.15 ± 0.07			
Palmitic acid 16:0	**22.78 ± 2.65**	23.59 ± 1.85*	23.44 ± 1.88	22.72 ± 2.19	23.52 ± 1.86	22.68 ± 1.79		**0.013**	
Margaric acid 17:0	**0.37 ± 0.7**	0.34 ± 0.12*	0.32 ± 0.09	0.3 ± 0.14	0.35 ± 0.16	0.36 ± 0.09			
Stearic acid 18:0	8.56 ± 3.03	8.14 ± 1.26	7.81 ± 1.39	8.25 ± 1.08	8.47 ± 1.09	8.91 ± 1.39		**0.015**	
Arachidic acid 20: 0	**0.13 ± 0.16**	0.19 ± 0.13*	0.16 ± 0.14	0.14 ± 0.15	0.20 ± 0.12	0.21 ± 0.13			
Behenic acid 22:0	0.82 ± 0.22	0.86 ± 0.11	0.84 ± 0.14	0.92 ± 0.14^b^	0.88 ± 0.07	0.9 ± 0.14		**0.018**	
Lignoceric acid 24:0	0.64 ± 0.37	0.77 ± 0.23	0.8 ± 0.24	0.88 ± 0.24	0.74 ± 0.25	0.76 ± 0.22			
**MUFA**	22.29 ± 3.59	22.91 ± 2.74	23.39 ± 3.05	**21.94 ± 2.37**	22.04 ± 2.24	**20.70 ± 1.57^a^**	**0.029**	**0.004**	
Myristoleic acid 14:1	0.01 ± 0.03	0.007 ± 0.02	0.003 ± 0.01	0.004 ± 0.01	0.01 ± 0.02	0.01 ± 0.01			
Palmitoleic acid 16:1 n-7	1.01 ± 0.49	0.98 ± 0.35	1.00 ± 0.33	0.94 ± 0.26	**0.99 ± 0.37**	**0.81 ± 0.28^b^**			
Oleic acid 18:1 n-9	20.03 ± 3.36	20.63 ± 2.54	**21.05 ± 2.89**	**19.7 ± 2.29^b^**	**19.80 ± 2^a^**	**18.52 ± 1.34^a,b^**	**0.027**	**0.005**	
Eicosenoic acid 20:1	0.01 ± 0.03	0.01 ± 0.01	0.001 ± 0.01	**0**	0.01 ± 0.02	**0.01 ± 0.03** ^a^	**0.01**		
Nervonic 24:1 n-9	1.22 ± 0.43	1.27 ± 0.3	1.33 ± 0.24	1.28 ± 0.30	1.21 ± 0.38	1.34 ± 0.38			
**PUFA n-6**	**40.62 ± 4.75**	38.68 ± 2.83*	38.42 ± 3.44	39.64 ± 3.46	38.93 ± 1.72	39.39 ± 3.49			
Linoleic acid (LA) 18:2 n-6	**31.49 ± 4.41**	29.69 ± 2.99*	29.61 ± 3.57	30.87 ± 3.51	29.91 ± 2.02	30.32 ± 3.67			
Gamma-linolenic acid 18:3 n-6	0.35 ± 0.24	0.28 ± 0.22	0.24 ± 0.25	0.26 ± 0.2	**0.30 ± 0.22**	**0.21 ± 0.15^b^**			**0.044**
Eicosadienoic acid 20:2 n-6	**0.17 ± 0.16**	0.25 ± 0.14*	0.20 ± 0.14	0.19 ± 0.16	0.26 ± 0.12	0.21 ± 0.13			
Dihomo-γ-linolenic acid (DGLA) 20:3 n-6	1.61 ± 0.44	1.57 ± 0.3	1.56 ± 0.22	**1.62 ± 0.45**	**1.56 ± 0.22**	**1.16 ± 0.28^a,b^**	**0.04**	**0.008**	**0.001**
Arachidonic acid (AA) 20:4 n-6	6.99 ± 1.33	6.87 ± 1.07	6.79 ± 1.05	6.72 ± 1.05	6.87 ± 1.14	6.88 ± 0.84			
**PUFA n-3**	2.89 ± 0.92	3.27 ± 1.26	3.43 ± 1.36	**3.23 ± 0.99**	**3.34 ± 1.14**	**6.27 ± 2.50^a,b^**	** < 0.001**	**<0.001**	** < 0.001**
Alfa-linolenic acid 18:3 n-3	0.35 ± 0.39	0.31 ± 0.21	0.27 ± 0.18	0.22 ± 0.17	0.37 ± 0.25	0.33 ± 0.22			
Eicosapentaenoic acid (EPA) 20:5 n-3	0.26 ± 0.24	0.40 ± 0.44	0.44 ± 0.45	**0.28 ± 0.21**	**0.35 ± 0.33**	**1.21 ± 0.75^a,b^**	** < 0.001**	**0.003**	** < 0.001**
Docosapentaenoic acid 22:5 n-3	0.52 ± 0.26	0.6 ± 0.18	0.58 ± 0.13	0.58 ± 0.21	0.60 ± 0.20	0.58 ± 0.24			
Docosahexaenoic acid (DHA) 22:6 n-3	1.75 ± 0.71	1.77 ± 0.69	1.89 ± 0.75	**1.87 ± 0.74**	**1.76 ± 0.64**	**3.82 ± 1.05^a,b^**	** < 0.001**	**<0.001**	** < 0.001**
**PUFA**	**43.52 ± 4.64**	41.40 ± 4.19*	41.23 ± 3.58	43.15 ± 4.13	**41.61 ± 4.88**	**45.11 ± 12.95^b^**		**0.046**	
**PUFA > 18C n-6**	8.78 ± 1.56	8.7 ± 1.17	8.56 ± 1.19	8.5 ± 1.16	8.7 ± 1.15	8.31 ± 0.96	** < 0.001**	**<0.001**	** < 0.001**
**PUFA > 18C n-3**	2.54 ± 0.83	2.78 ± 1.12	2.91 ± 1.24	**2.73 ± 0.93**	**2.73 ± 0.93**	**5.61 ± 1.83^a,b^**	** < 0.001**	**<0.001**	** < 0.001**
**SFA/MUFA**	1.59 ± 0.52	1.54 ± 0.23	1.49 ± 0.24	1.57 ± 0.21	1.6 ± 0.22	1.67 ± 0.13	**0.05**	**0.02**	
**MUFA/PUFA**	0.52 ± 0.10	0.58 ± 0.13	0.60 ± 0.14	**0.54 ± 0.13**	**0.55 ± 0.12**	**0.45 ± 0.18^a,b^**	**0.036**	**0.008**	
**Unsaturation index**	4.04 ± 0.78	3.81 ± 0.41	3.88 ± 0.44	**3.9 ± 0.42**	**3.76 ± 0.39**	**4.25 ± 0.48^a,b^**		**0.004**	**0.003**

*Comparison between baseline and after 6 months of treatment with DHA intervention or with placebo in ASD group.*

SFA, saturated fatty acids; MUFA, Monounsaturated fatty acids; PUFA, Polyunsaturated fatty acid. “Results are expressed as percentages of total fatty acids except for the unsaturation index which was obtained as the sum of the percentages of each fatty acid multiplied by the corresponding number of double bonds.” *p < 0.05 value between reference vs. ASD total. ^a^p < 0.05 value between intergroup; ^b^p < 0.05 value between intragroup.

*The values of p for “intervention” are the “intergroup” differences. The Time is the difference between times considering both groups of intervention at the same time (not separately).*

*The column “Time × Intervention” gives the difference “intergroups” adjusted by time. Bold font indicates statistical significance.*

**TABLE 4:** Comparison of fatty acid profile in erythrocytes profile between reference group and ASD children.

Fatty acids (%)			Placebo n:25		Intervention n:19		*P*-value		
	Reference N:59	ASD total N:54	Baseline	Final intervention	Baseline	Final intervention	Intervention	Time	Time x intervention
**SFA**	49.08 ± 3.97	48.4 ± 2.35	47.79 ± 2.02	47.61 ± 2.19	48.64 ± 2.27	49.02 ± 3.25			
Myristic acid 14:0	**0.21 ± 0.1**	0.25 ± 0.08*	**0.26 ± 0.08**	**0.20 ± 0.08^b^**	0.25 ± 0.08	0.25 ± 0.08		**0.032**	
Palmitic acid 16:0	**28.94 ± 3.83**	27.71 ± 3.46*	27.72 ± 3.09	27.35 ± 3.10	**26.91 ± 3.07**	**28.03 ± 3.77** ^b^			**0.032**
Margaric acid 17:0	**0.19 ± 0.05**	0.21 ± 0.04*	0.21 ± 0.04	0.21 ± 0.05	0.23 ± 0.04	0.23 ± 0.05			
Stearic acid 18:0	15.54 ± 2.17	15.59 ± 1.49	**15.13 ± 1.4**	15.33 ± 1	**16.37 ± 1.39^a^**	15.99 ± 1.62	**0.014**		
Arachidic acid 20: 0	**0.29 ± 0.11**	0.35 ± 0.13*	0.31 ± 0.09	0.32 ± 0.08	0.35 ± 0.1	0.33 ± 0.11			
Behenic acid 22:0	1.11 ± 0.34	1.2 ± 0.29	1.15 ± 0.3	1.13 ± 0.25	1.24 ± 0.28	1.19 ± 0.31			
Lignoceric acid 24:0	2.76 ± 0.89	3.06 ± 0.84	2.98 ± 0.77	3.05 ± 0.78	3.26 ± 0.90	2.97 ± 0.81			
**MUFA**	17.3 ± 2.35	17.72 ± 1.78	18.15 ± 1.43	17.97 ± 1.53	17.61 ± 1.69	17.39 ± 1.22			
Palmitoleic acid 16:1 n-7	**0.19 ± 0.12**	0.21 ± 0.09*	0.23 ± 0.08	0.20 ± 0.08	0.20 ± 0.08	0.21 ± 0.06			
Oleic acid 18:1 n-9	14.21 ± 2	14.09 ± 1.72	14.42 ± 1.3	14.28 ± 1.42	13.76 ± 1.68	13.67 ± 1.43			
Nervonic 24:1 n-9	**2.90 ± 0.94**	3.41 ± 0.95*	3.5 ± 0.96	3.48 ± 0.91	3.65 ± 0.89	3.51 ± 0.91			
**PUFA n-6**	28.29 ± 4.65	27.1 ± 2.69	27.27 ± 2.83	**28.09 ± 2.05**	**27.08 ± 2.42**	**24.83 ± 2.20^a,b^**	**0.005**		**0.001**
Linoleic acid (LA) 18:2 n-6	13.13 ± 5.11	11.93 ± 2.43	12.15 ± 2.38	**12.7 ± 1.88**	12.1 ± 2.39	**11.6 ± 1.52^a^**			
Eicosadienoic acid 20:2 n-6	0.19 ± 0.08	0.2 ± 0.05	0.20 ± 0.05	0.20 ± 0.05	0.21 ± 0.05	0.22 ± 0.08			
Dihomo-γ-linolenic acid (DGLA) 20:3 n-6	**1.08 ± 0.30**	1.19 ± 0.25*	1.2 ± 0.25	1.16 ± 0.26	**1.16 ± 0.25**	**1 ± 0.30** ^b^		**0.05**	
Arachidonic acid (AA) 20:4 n-6	13.88 ± 2.29	13.58 ± 1.89	13.70 ± 1.85	**14.02 ± 1.69**	**13.61 ± 1.77**	**11.99 ± 1.35^a,b^**	**0.025**	**0.038**	**0.001**
**PUFA n-3**	5.3 ± 4.07	5.87 ± 1.89*	5.97 ± 1.91	**5.76 ± 1.65**	**6.27 ± 1.84**	**8.97 ± 2.19^a,b^**	**0.001**	**0.001**	**0.001**
Eicosapentaenoic acid (EPA) 20:5 n-3	**1.27 ± 3.83**	1.01 ± 1.03*	0.82 ± 0.7	0.92 ± 1.02	1.23 ± 1.32	1.51 ± 1.45	**0.042**		
Docosapentaenoic acid 22:5 n-3	**1.10 ± 0.44**	1.32 ± 0.36*	**1.38 ± 0.38**	**1.23 ± 0.34^b^**	1.36 ± 0.31	1.33 ± 0.34		**0.032**	
Docosahexaenoic acid (DHA) 22:6 n-3	**2.92 ± 1.04**	3.52 ± 1.25*	3.75 ± 1.4	**3.59 ± 1.4**	**3.66 ± 1.06**	**6.12 ± 1.4^a,b^**	**0.002**	**<0.001**	**<0.001**
**PUFA**	33.6 ± 4.88	32.79 ± 2.83	33.24 ± 1.96	34.86 ± 1.87	33.36 ± 2.49	33.8 ± 2.57			
**PUFA > 18C n-6**	14.97 ± 2.25	14.78 ± 1.86	14.9 ± 1.79	**15.39 ± 1.38**	**14.7 ± 1.82**	**13 ± 1.42^a,b^**	**0.005**		**0.000**
**PUFA > 18C n-3**	5.3 ± 4.07	5.87 ± 1.89*	5.97 ± 1.91	5.76 ± 1.65	**6.27 ± 1.84**	**8.97 ± 2.19^a,b^**	**0.001**	**0.001**	**0.001**
**SFA/MUFA**	2.89 ± 0.46	2.76 ± 0.35	2.65 ± 0.29	2.67 ± 0.31	2.78 ± 0.32	2.83 ± 0.29			
**MUFA/PUFA**	0.52 ± 0.09	0.54 ± 0.07	0.54 ± 0.05	0.53 ± 0.06	0.53 ± 0.06	0.51 ± 0.05			

*Comparison between baseline and after 6 months of treatment with DHA intervention or with placebo in ASD group.*

*SFA, saturated fatty acids; MUFA, Monounsaturated fatty acids; PUFA, Polyunsaturated fatty acid. Results are expressed as percentages of total fatty acids except for the unsaturation index, which was obtained as the sum of the percentages of each fatty acid multiplied by the corresponding number of double bonds.*

*p < 0.05 value between reference vs. ASD total. ^a^p < 0.05 value between intergroup; ^b^p < 0.05 value between intragroup. The values of p for “intervention” are the “intergroup” differences. The Time is the difference between times considering both groups of intervention at the same time (not separately). The column “Time × Intervention” gives the difference “intergroups” adjusted by time. Bold font indicates statistical significance.

At baseline, children with ASD had significantly higher plasma percentages of palmitic acid (16:0) (*p* = 0.011) and total saturated fatty acids (SFA) (*p* = 0.043) and the lower percentages of linoleic acid (18:2 *n*-6) (*p* = 0.012) and total PUFA n-6 (*p* = 0.009). The percentages of the minority acids, pentadecanoic (15:0) (*p* = 0.008), and eicosadienoic (20:2 n-6) (*p* = 0.013) were higher in ASD than in the reference children (Table 3).

After 6 months of dietary intervention, the ASD group intervened with the DHA enriched product significantly increased the plasma percentage of DHA (*p* = 0.001) and those of n-3 PUFA (*p* < 0.001) and total PUFA (*p* = 0.003). Both the placebo and the intervened ASD groups decreased the percentage of oleic acid. However, the ratio MUFA/PUFA decreased significantly only in the ASD group with DHA intervention. Concerning the interaction of treatment per time, only γ-linolenic acid (GLA) (18:3 n-6) and dihomo-gamma-linolenic acid (DGLA) (20:3 n-6) decreased significantly in the ASD group of intervention with DHA, with an increase in EPA and DHA.

In the analysis of erythrocyte fatty acids at baseline, minor but significant differences for the FA profiles were found between the reference and the ASD group, characterized by the lower percentages of palmitic acid (16:0) (*p* = 0.027) and EPA (*p* = 0.02), and higher of palmitoleic acid (16:1 n-7) (*p* = 0.04), margaric acid (17:00) (*p* = 0.01), docosapentaenoic acid (DPA) (22:5 n-3) (*p* = 0.004) and DHA (*p* = 0.014) in the ADS group. After the intervention in the ASD group that received DHA, the levels of this FA significantly rose (3.66 ± 1.06% vs. 6.12 ± 1.4%, *p* < 0.001) and palmitic acid (*p* = 0.033) and EPA (*p* = 0.042) also increased, while stearic acid (18:0) and arachidonic acid (AA) (20:4 n-6) decreased. So, the increased levels of n-3 PUFA and decreased levels of n-6 PUFA were found, while the unsaturation index was fairly maintained. About the interaction of treatment per time, only palmitic acid, DHA (both with an increase), and AA (a decrease) showed significant differences.

Parents were called every 2 months, and no adverse effects were found in children during the intervention with DHA or placebo. An adequate tolerance and adherence were registered in the 54 children until the trial finished.

### Plasma Cytokines

Comparing plasma cytokines at baseline between the reference and ASD groups, the higher levels of NGF were detected in ASD children as has been previously reported by our group (40).

Table 5 presents the plasma cytokines in children with ASD at baseline and after 6 months of treatment with the DHA supplement or placebo. No major changes were observed for plasma cytokines due to the treatment or interaction of treatment per time. Nevertheless, from baseline to the final intervention, NCAM plasma levels increased in both the placebo and the group with DHA supplement. A significant increase in cathepsin D (*p* = 0.002) and ICAM (*p* = 0.047) was observed in the group that received treatment. No significant correlations between the plasma levels of cytokines and fatty acids in plasma and erythrocytes were observed either at baseline or after the intervention.

**TABLE 5:** Plasma cytokines levels in children with ASDs at baseline and after 6 months with DHA intervention or placebo.

Placebo				Intervention			*P*-value
Plasma cytokines	N	Baseline	Final intervention	N	Baseline	Final intervention	Time
BDNF (μg/L)	25	5.84 ± 4.13	5.32 ± 4.42	19	5.92 ± 5.48	5.22 ± 3.46	
Cathepsine D (μg/L)	25	167.28 ± 174.22	195.51 ± 65.46	**19**	**106.64 ± 42.35**	** ** 223.62 ± 139.12^b^	**0.008**
MPO (μg/L)	25	160.03 ± 269.59	111.20 ± 96.88	18	201.42 ± 285.51	136.85 ± 141.59	
NCAM (μg/L)	**25**	**388.41 ± 63.58**	**427.05 ± 82.06^b^**	**19**	**372.67 ± 73.01**	**430.69 ± 85.69^b^**	**0.001**
PAI-1 (μ/L)	25	79.55 ± 52.62	71.39 ± 31	19	67.128 ± 41.21	77.57 ± 34.79	
RANTES (μg/L)	25	80.18 ± 54.58	69.67 ± 45.73	19	73.02 ± 53.55	67.21 ± 55.69	
ICAMs (μg/L)	**25**	170.75 ± 33.59	177.56 ± 39.85	**19**	**166.03 ± 34.05**	**189.45 ± 53.05^b^**	**0.07**
VCAMs (μg/L)	25	1071.70 ± 179.36	1,153 ± 220.45	19	1048.77 ± 275.36	1153.46 ± 321.78	**0.028**
HGF (pg/ml)	25	172.77 ± 62.29	155.85 ± 50.77	19	186.65 ± 81.74	171.61 ± 80.95	
IL6 (pg/ml)	7	2.54 ± 2.83	1.3 ± 1.14	8	2.57 ± 1.85	4.12 ± 4.70	
IL8 (pg/ml)	22	2.46 ± 2.71	1.83 ± 2.37	19	3.76 ± 3.59	4.21 ± 5.58	
MCP (pg/ml)	25	132.62 ± 73.86	**111.15 ± 31.67**	19	142.59 ± 62.51	**135.56 ± 46.04^a^**	
NGF (pg/ml)	4	8.52 ± 8.20	10.17 ± 13.12	8	11.62 ± 9.04	11.98 ± 13.26	
TNFα (pg/ml)	25	3.25 ± 1.53	3.33 ± 0.88	19	3.52 ± 1.49	3.51 ± 1.32	

*BDNF, brain-derived neurotrophic factor; MPO, myeloperoxidase; NCAM, neuronal cell adhesion molecule; PAI, plasminogen activation inhibitory factor; 1 RANTES, regulated on activation normal T cell expressed and secreted; ICAM, intercellular adhesion molecule; VCAM, vascular cell adhesion molecule; HGF, hepatocyte growth factor; IL, interleukin; MCP-1, monocyte chemotactic protein; NGF, nerve growth factor; TNFα, tumor necrosis factor α.*

*Data are given as the mean ± SD. ^a^p < 0.05 value between intergroup ^b^p < 0.05 value between intragroup. Bold font indicates statistical significance.*

*The value of p for intervention and Time × intervention in all variables were non-significant. The values of p for “intervention” are the “intergroup” differences. The Time is the difference between times considering both groups of intervention at the same time (not separately). The column “Time × Intervention” gives the difference “intergroups” adjusted by time.*

### Clinical Test for Autism Spectrum Disorder Children Evaluation

*En general, incrementoe en los puntajes de los test aplicados implica empeoramiento de los sintomas, excepto en las distintas areas del test de Battelle donde es a la inversa.*

The principal results of the ASD test are presented in Table 6. No differences were observed in the test scores between the two groups of ASD before the intervention. After 6 months of intervention, there was a significant decrease in the CARS score in both groups. When repeating the SDQ tests at 6 months, a significant decrease was observed in the areas of emotional symptoms (*p* = 0.04), conduct problems (*p* = 0.02), and impact on quality (*p* < 0.001) of life in ASD group with PUFAs treatment, but always in the range for ASD children score. However, in the area that assesses the main characteristic of autism for this group, we did not detect differences in the pro-social behavior and relationship with other children. When applying the Battelle test to both groups at 6 months, a significant improvement was observed in the areas of receptive (*p* = 0.001), expression *p* = 0.017) and communication (*p* = 0.001), in the placebo ASD group. In the different areas of assessment with the PDDBI test, only an increase in the “social approach behaviors” area in both groups was observed. No significant correlations were observed between plasma and erythrocyte FA levels with test scores or with FA intakes (data not shown).

**TABLE 6:** Clinical test for ASD children evaluation at baseline and after 6 months with DHA intervention or placebo.

		Placebo n:25		Treatment n:19		*P*-value		
TEST	AREAS	Baseline	Final intervention	Baseline	Final intervention	Intervention	Time	Time × Intervention
**CARS**		**33.01 ± 6.84**	**28.98 ± 5.48^b^**	**32.22 ± 8.68**	**28.69 ± 7.74^b^**		**<0.001**	
**SDQ**	Emotional symptoms	1.5 ± 1.9	1.6 ± 1.6	**1.8 ± 1.5**	**1.2 ± 1.4^b^**			
	Conduct problems	2.5 ± 1.7	2.4 ± 1.6	**3.5 ± 2**	**2.6 ± 1.3^b^**			
	Hyperactivity	7.2 ± 2.4	7 ± 2.1	7.1 ± 2.1	6.5 ± 2.6	NS		
	Relations with Peers	5.3 ± 2	5 ± 1.6	5.4 ± 1.6	4.8 ± 1.7			
	Prosocial behavior	**2.8 ± 1.5**	**3.5 ± 2.2^b^**	4.1 ± 2.9	3.8 ± 2.4			
	Impact	3 **±** 2.1	2.48 ± 2.1	**3.9 ± 2.6**	**1.7 ± 2.4^b^**		**0.002**	**0.033**
**BATTELLE**	Personal/social	43.8 ± 13.9	50.1 ± 20.4	43.8 ± 16.4	41.7 ± 20.8			
	Adaptive	53.9 ± 14.1	49.4 ± 19.2	48.8 ± 16.2	45.2 ± 18.1			
	Gross motor	72.2 ± 17.2	66.6 ± 25.5	61.8 ± 13.7	55.6 ± 20			
	Fine motor	69.3 ± 16.9	67.45 ± 22.5	64.5 ± 20.7	59.4 ± 20.8			
	Total motor	70.4 ± 12.5	68.64 ± 20.2	65.4 ± 15.6	60.4 ± 21.4	NS		
	Receptive	48.6 ± 22.3	**60.20 ± 23**	44.9 ± 25.9	**42.9 ± 25.3^a^**		**0.027**	**0.007**
	Expressive	**44.8 ± 18.2**	**55.6 ± 25.3^b^**	47.6 ± 24.1	44.2 ± 27.4			**0.032**
	Communication	**44.5 ± 16.7**	**57.9 ± 24.5^b^**	44.7 ± 23.2	43.2 ± 25.3		**0.014**	**0.007**
	Cognitive	75.8 ± 20.1	**79 ± 28.8**	68.9 ± 24.4	**60.7 ± 21.8**^a^			
	Battelle total	57.8 ± 13.2	62 ± 21.6	54.3 ± 16.3	52.3 ± 21.8			
**PDDBI**	Sensory	49.6 ± 9.2	47 ± 7.9	51.9 ± 12.3	51.4 ± 12.3			
	Ritual score	52.4 ± 8.9	51.8 ± 8.1	52.7 ± 11.8	49.6 ± 10.6			
	Social pragmatic problems	47.5 ± 9.1	48.1 ± 6.4	47.8 ± 8.8	47.9 ± 7.5			
	Semantic pragmatic problems	49.3 ± 9.9	49.3 ± 9.4	47.4 ± 9.3	48.9 ± 10.1	NS		
	Social approach behaviors	**52.1 ± 9.9**	**55.4 ± 8.9^b^**	**48.8 ± 11.7**	**51.9 ± 11.6^b^**		**0.001**	
	Expressive language	51.19 ± 10.38	53.54 ± 11.93	51.7 ± 12.6	52.3 ± 13.3			
	Score autism	**48.96 ± 8.857**	**46.38 ± 7.161^b^**	49.3 ± 12.4	48.3 ± 12.9		0.05	

*CARS, Childhood Autism Rating Scale Test; SDQ, Strengths and Difficulties Questionnaire; PDDBI, Pervasive Developmental Disorders Behavior Inventory.*

*Data are given as the mean ± SD. ^a^p < 0.05 value between intergroup; ^b^p < 0.05 value between intragroup.*

*NS, non-significant. The values of p for “intervention” are the “intergroup” differences. The Time is the difference between times considering both groups of intervention at the same time (not separately). The column “Time × Intervention” gives the difference “intergroups” adjusted by time. Bold font indicates statistical significance.*

## Discussion

In the present study, at baseline, the FA profiles of both plasma and erythrocytes in this cohort of Spanish ASD children can be considered within the normal ranges without major differences compared with the healthy reference group. After 6 months of supplementation with 800 mg/day of DHA there was an increase in DHA levels in plasma and erythrocytes, as expected, without changes in the clinical evaluation associated with this treatment. We observed a high adherence to the treatment without any serious adverse outcomes and even a beneficial decrease in triacylglycerol plasma concentrations as has been reported in the use of n-3 LC-PUFA supplements in other adult pathologies (41).

There are no adequate standardized ranges for FA concentrations or percentages in children. Petersen et al. refer, for children between 0 and 3 years old, healthy levels for DHA about 2–4%, similarly as other authors have reported (42, 43). Moreover, Drover et al. indicated that a DHA-standard concentration of 0.32% ensures an adequate cognitive function (44). Indeed, in our sample of ASD children, there are no deficiencies related to FA that could contribute to the origin or evolution of this pathology. This result discards the hypothesis suggested by some authors about the alterations of FA concentrations in patients with ASD, especially related to a deficit in DHA if children have a regular intake of fish and shellfish as it occurred in our Spanish sample. In addition, it should be considered that during pregnancy or in infants under 2 years of age, there might be an omega-3 PUFA deficiency associated with the lower intakes of those fatty acids and this could also contribute to brain dysfunction later in life; however, this situation would not be directly related to ASD (45). A meta-analysis (46) of fifteen case-control studies (*n* = 1,193), most of them were done in children younger than 12 years and most using an intake of EPA and DHA ranged from 0.70 to 0.84 g/day and 0.24 to 0.70 g/day, respectively, found that, compared with typically developed individuals, the ASD group had lower plasma EPA, DHA, and AA, without significant reduction in the total n-6 PUFAs and a lower ratio of total n-3 to total n-6 fatty acids. These relative deficiencies have been justified by digestive or microbiota alterations as deficiencies in digestive enzymes (20) or an increase in intestinal permeability (“leaky gut”) associated with gastrointestinal (GI) inflammation. Low-mild gut inflammation and augmented intestinal permeability have been reported in ASD children associated with dysbiosis and GI symptoms (21). However, altered intestinal permeability could also be related to alteration in the plasma membrane of enterocytes due to a potential derangement of their fatty acid profile and essential fatty acid and long-chain PUFA deficiency.

Our results at the basal time show normal concentrations in DHA/EPA with some significant differences between the percentage of some plasma and erythrocyte FA in children with ASD compared with those of reference. Nevertheless, some of them are little quantitatively assessable, such as pentadecanoic acid 15:0, margaric acid 17:0, arachidic acid 20:0, and eicosadienoic acid 20:2 n-6, as their contents are very low and the interpretation to establish clinical changes is difficult. Nevertheless, these could be explained in part by the highly selective eating patterns of these children with ASD. Relevant data after the evaluation of the intake in these children have been recently published by our group (33). In fact, the decrease in linoleic acid, which has already been reported in other cohorts of autistic children (47, 48) would be justified by the high intake of dairy products, which in turn explain the relative increase in the percentage of some saturated fatty acids, e.g., palmitic acid, and odd FA, namely, margaric acid.

The role of FA in the membranes of different cells and in the neurotransmitters supports the hypothesis that DHA supplements would improve the symptoms of ASD (15). The different mechanisms of involvement of n-3 PUFA levels in the etiology of ASD have been suggested as defects in enzymes involved in the DHA and EPA production from α-linolenic acid (ALA), known as FA desaturases (FADS), by deficiencies in its process of cell membrane incorporation, or an alteration in its metabolism, e.g., through a possible dysfunction in mitochondrial PUFA oxidation (49, 50). These FA alterations would justify the higher incidence of ASD in boys, because girls appear to have a higher conversion rate of ALA into DHA, associated with the higher hepatic expression of PUFA desaturase enzymes, and probably longer DHA half-life in plasma compared with boys (51). However, in this study, n-3 PUFA normal levels in erythrocytes in ASD children seem to ensure a normal activity in membranes and in other cells.

The clinical trials with DHA supplementation in ASD are very heterogeneous, in age ranges and also in the doses used and the duration of the administration. Here, as the literature refers, the levels of 800 mg of DHA were used because lower levels do not seem to be effective (24) and with a duration of 6 months to reach steady-state levels in erythrocytes (52). Moreover, in our study there is an indirect verification of the intake of intervention oils, not only due to the elevation of plasma DHA and EPA confirmed in the n-3 LC-PUFA treated ASD group as reported by other authors (17, 53), but also the increase in erythrocytes reflecting the prolonged intake and the adherence during the 6 months without problems in the absorption or metabolism. In addition, Parellada et al. observed an improvement in the omega6/omega3 ratio in the erythrocyte membrane without modification in oxidation levels and clinical improvement associated with this treatment in ASD children (54).

Here, after 6 months of treatment, the ASD group intervened with the DHA enriched product, increased the plasma percentage of DHA and those of total PUFA and PUFA n-3 that contributed to an enhanced unsaturation index (concentration of the unsaturated product proportional to the sum of the unsaturated product and the saturated substrate) in a balanced way. Nevertheless, the percentage of AA was lower in the erythrocyte for the DHA intervened group suggesting that the consumption of the DHA supplement would limit the synthesis and/or incorporation of AA to cell membranes. In this regard, it is well known that increased LC-PUFA inhibit the delta-6 and delta-5 FA desaturases. As AA is a key compound in the synthesis of eicosanoids and infant growth, an excessive intake of DHA might compromise the appropriate development of ASD children (55, 56).

The efficacy of DHA supplementation has been measured mainly by assessing the modifications of the symptoms of ASD through validated tests (15–17). The results obtained in the present study show that there is no clinical improvement after treatment with DHA/EPA. Although the subscales of the SDQ showed “friendly and helpful behavior” and “impact on the child’s life” improved in the ASD children who received the treatment; however, in certain areas of the Battelle test (receptive, expressive, and total items), the improvement was in the placebo group. So, these data should be interpreted with caution. DHA treatment does not seem to be directly related to the evolution in the central symptoms of autism and that changes in certain areas may be due to the continuous learning strategies and psychotherapies from both patients and parents. In fact, these ASD children are subjected to intensive behavioral therapies after the diagnosis, so a little progression in psychomotor development is expected, but not directly associated with this therapy with PUFAs if there are no significant differences between the groups in blood levels. Therefore, other factors can intervene in the improvement of these variables over time. There are a number of non-quantifiable biases in these studies related to the behavioral therapies applied to these children dependent on family or support teams that cannot be avoided in quality although they do not solve the origin of problem (3, 57, 58).

Besides, although some studies have reported a little improvement in hyperactivity symptoms in ASD patients treated with DHA, in this study, there were no relevant differences in scores in the PDDBI, SDQ, and CARS scales after the trial to find differential clinical responses. In fact, in contrast with other clinical trials and meta-analyses that assessed the efficacy of omega-3 FA in hyperactivity in ASD (59, 60), in our study, there was no statistical significance in the subscale of the SDQ-hyperactivity.

Eicosapentaenoic and DHA have immunomodulatory, anti-inflammatory, and anti-oxidant properties, and both have important roles in brain function and structure and the neurotransmitter system (61). In fact, the increase in the prevalence of ASD has been partly justified by the variation in the dietary profiles of the population with an increase in dietary pro-inflammatory omega-6 PUFA, through meat and processed food, and a decrease in anti-inflammatory omega-3 PUFA, present mainly in seafood. There are already several studies which suggested that a low proinflammatory status could contribute to the pathogenesis of ASD (62, 63). Moreover, changes in inflammatory cytokine levels in patients with ASD by administering PUFA or associations with the clinical evolution have been scarcely evaluated. Only Bent et al. reported in a clinical trial a negative correlation between IL-2 and hyperactivity (17), while other authors (16) did not find associations with the PUFA treatment.

In the present work, different plasma cytokines were measured in ASD children before and after the treatment with DHA. We did not observe changes in cytokine levels due to treatment, although an increase in cathepsin and ICAM was shown after 6 months of the trial, which can be justified by the passage of time, because of the increment in both groups although it was significantly higher in the ASD group. The role of ICAM-1 in driving the inflammatory response and the newly emerging roles of ICAM-1 in epithelial injury-resolution responses, as well as immune cell effector function in inflammation and tumorigenesis, have been described. Cathepsin D is related to apoptosis and elevations have been detected in the brain of autistic subjects, suggesting a relationship between this molecule and ASD pathogenesis (64, 65). However, it is difficult to determine the significance of the elevation of these two molecules in this study and its possible relationship with the disease.

The principal limitation of this work is the sample size since it is difficult to carry out nutritional interventions in children with ASD for a long time and to take blood samples for analysis. In fact, analysis by sex was not performed since, considering that ASD is highly prevalent in boys (83%), a subdivision by sex would have implied taking larger samples. Moreover, the sample size could have conditioned the failure to find relevant differences in secondary outcomes, such as ASD clinical scores and plasma cytokines. However, the sample is having been adequate according to the estimation based in the principal variable for statistical analysis. In the statistical calculation, a loss of follow-up rate of 5% was estimated and the primary method of intention-to-treat was used to avoid biases for patient dropout. A sample from only one center for the present trial, using a fixed dose for DHA and time of administration, and a determined battery of tests (not especially based on hyperactivity) and biomarkers measurements, are some other limitations for this study.

Methods are different from other trials making it difficult to compare all these results in a future meta-analysis. Notwithstanding, patients with ASD were selected younger than 7 years to maximize the effect of the major neuronal plasticity in this stage of development. Moreover, there was a homogeneus pediatric sample, with a well-matched age range and sex distribution, and with a rigorous method in the supplementation and analysis for concentrations in plasma and erythrocyte FA profile as the principal strengths. Possible biases between the two randomized groups have been the controlled monitoring levels of FA and the scores in the psychological testing with no differences between them.

There are often contradictory results in the published clinical trials. Thus, there seems to be insufficient evidence to recommend omega-3 FA supplementation to all children with ASD. Based on anecdotal experiences on non-randomized trials, we cannot exclude that there might be a subset of patients with ASD who could need these supplements. About omega-3 FA in ASD children studies, it is neccesary to consider appropriate doses, time for treatment, and biochemical biomarkers, to evaluate the effects in metabolism, and adequate tests to discriminate the evolution in neurological and psychological items in children with this pathology.

## Conclusion

In the present study, children with ASD exhibit an appropriate omega-3 FA status. The intervention for 6 months with a DHA supplement, despite increasing plasma and erythrocyte DHA, did not improve the tests of evaluation of ASD nor did modify the levels of plasma cytokines. Dietary intervention with DHA in childhood ASD was not effective; therefore, it must be very cautious with the prescription of these dietary supplements, without a confirmed deficiency or altered metabolism in specific biomarkers.

## Data Availability Statement

The raw data supporting the conclusions of this article will be made available by the authors, without undue reservation.

## Ethics Statement

The studies involving human participants were reviewed and approved by the ethics committee of Corboba. Written informed consent to participate in this study was provided by the participants’ legal guardian/next of kin.

## Author Contributions

MT-A, AG-F, MG-C, and JP-N contributed to the study conception and study design. MT-A, MG-C, and AG were responsible for the interpretation of the data, as well as drafting the manuscript. KF-R, MO, and MM collected all the data, acquired the behavioral data, and carried out the analysis. PM-B selected the ASD patients and perform all the tests. All authors read and approved the final manuscript.

## Conflict of Interest

MO was a worker of Biosearch Life, a company that marketed the placebo and the nutritional supplement, EUPOLY-3 DHA^®^ Infant, following the regulations of the European Union. These supplements were provided free of charge by Biosearch SA (Granada, Spain). The remaining authors declare that the research was conducted in the absence of any commercial or financial relationships that could be construed as a potential conflict of interest.

## Publisher’s Note

All claims expressed in this article are solely those of the authors and do not necessarily represent those of their affiliated organizations, or those of the publisher, the editors and the reviewers. Any product that may be evaluated in this article, or claim that may be made by its manufacturer, is not guaranteed or endorsed by the publisher.

## References

[1] Arlington VAA. Diagnostic and statistical manual of mental disorders. *Am Psychiatr Assoc.* (2013) 5:612–3.

[2] Stepanova E Dowling S Phelps M Findling RL. Pharmacotherapy of emotional and behavioral symptoms associated with autism spectrum disorder in children and adolescents. *Dialogues Clin Neurosci.* (2017) 19:395–402. 10.31887/DCNS.2017.19.4/rfindling 29398934 PMC5789216

[3] Parletta N Milte CM Meyer BJ. Nutritional modulation of cognitive function and mental health. *J Nutr Biochem.* (2013) 24:725–43. 10.1016/j.jnutbio.2013.01.002 23517914

[4] Lauritzen L Brambilla P Mazzocchi A Harsløf LBS Ciappolino V Agostoni C. DHA effects in brain development and function. *Nutrients.* (2016) 8:6. 10.3390/nu8010006 26742060 PMC4728620

[5] Schuchardt JP Huss M Stauss-Grabo M Hahn A. Significance of long-chain polyunsaturated fatty acids (PUFAs) for the development and behaviour of children. *Eur J Pediatr.* (2010) 169:149–64. 10.1007/s00431-009-1035-8 19672626

[6] Agostoni C Nobile M Ciappolino V Delvecchio G Tesei A Turolo S The role of omega-3 fatty acids in developmental psychopathology: a systematic review on early psychosis, autism, and ADHD. *Int J Mol Sci.* (2017) 18:2608. 10.3390/ijms18122608 29207548 PMC5751211

[7] Chiang N Serha CN. Specialized pro-resolving mediator network: an update on production and actions. *Essays Biochem.* (2020) 64:443–62. 10.1042/EBC20200018 32885825 PMC7682745

[8] Bazan NG. Neuroprotectin D1-mediated anti-inflammatory and survival signaling in stroke, retinal degenerations, and Alzheimer’s disease. *J Lipid Res.* (2009) 50:S400–5. 10.1194/jlr.R800068-JLR200 19018037 PMC2674685

[9] Serhan CN Petasis NA. Resolvins and protectins in inflammation resolution. *Chem Rev.* (2011) 111:5922–43. 10.1021/cr100396c 21766791 PMC3192290

[10] Bozzatello P Rocca P Mantelli E Bellino S. Polyunsaturated fatty acids: what is their role in treatment of psychiatric disorders? *Int J Mol Sci.* (2019) 20:5257. 10.3390/ijms20215257 31652770 PMC6862261

[11] DeFilippis M. The use of complementary alternative medicine in children and adolescents with autism spectrum disorder. *Psychopharmacol Bull.* (2018) 48:40–63. 29382959 10.64719/pb.4560 PMC5765434

[12] Nath D. Complementary and alternative medicine in the school-age child with autism. *J Pediatr Heal Care.* (2017) 31:393–7. 10.1016/j.pedhc.2016.12.001 28017488

[13] Hopf KP Madren E Santianni KA. Use and perceived effectiveness of complementary and alternative medicine to treat and manage the symptoms of autism in children: a survey of parents in a community population. *J Altern Complement Med.* (2016) 22:25–32. 10.1089/acm.2015.0163 26654976 PMC4739350

[14] Croall ID Hoggard N Hadjivassiliou M. Gluten and autism spectrum disorder. *Nutrients.* (2021) 13:572. 10.3390/nu13020572 33572226 PMC7915454

[15] Amminger GP Berger GE Schäfer MR Klier C Friedrich MHFM. Omega-3 fatty acids supplementation in children with autism: a double-blind randomized, placebo-controlled pilot study. *Biol Psychiatry.* (2007) 61:551–3. 10.1016/j.biopsych.2006.05.007 16920077

[16] Mankad D Dupuis A Smile S Roberts W Brian J Lui T A randomized, placebo controlled trial of omega-3 fatty acids in the treatment of young children with autism. *Mol Autism.* (2015) 6:18. 10.1186/s13229-015-0010-7 25798215 PMC4367852

[17] Bent S Bertoglio K Ashwood P Bostrom A Hendren RL. A pilot randomized controlled trial of omega-3 fatty acids for autism spectrum disorder. *J Autism Dev Disord.* (2011) 41:545–54. 10.1007/s10803-010-1078-8 20683766 PMC3076562

[18] Bent S Hendren RL Zandi T Law K Choi JE Widjaja F Internet-based, randomized, controlled trial of omega-3 fatty acids for hyperactivity in autism. *J Am Acad Child Adolesc Psychiatry.* (2014) 53:658–66. 10.1016/j.jaac.2014.01.018 24839884 PMC4076340

[19] Yui K Koshiba M Nakamura S Kobayashi Y. Effects of large doses of arachidonic acid added to docosahexaenoic acid on social impairment in individuals with autism spectrum disorders: a double-blind, placebo-controlled, randomized trial. *J Clin Psychopharmacol.* (2012) 32:200–6. 10.1097/JCP.0b013e3182485791 22370992

[20] Kushak RI Lauwers GY Winter HS Buie TM. Intestinal disaccharidase activity in patients with autism: effect of age, gender, and intestinal inflammation. *Autism.* (2011) 15:285–94. 10.1177/1362361310369142 21415091

[21] Iovene MR Bombace F Maresca R Sapone A Iardino P Picardi A Intestinal dysbiosis and yeast isolation in stool of subjects with autism spectrum disorders. *Mycopathologia.* (2017) 182:349–63. 10.1007/s11046-016-0068-6 27655151

[22] Horvath A Łukasik J Szajewska H. ωV-3 fatty acid supplementation does not affect autism spectrum disorder in children: a systematic review and meta-analysis1-3. *J Nutr.* (2017) 147:367–76. 10.3945/jn.116.242354 28077731

[23] Cheng YS Tseng PT Chen YW Stubbs B Yang WC Chen TY Supplementation of omega 3 fatty acids may improve hyperactivity, lethargy, and stereotypy in children with autism spectrum disorders: a meta-analysis of randomized controlled trials. *Neuropsychiatr Dis Treat.* (2017) 13:2531–43. 10.2147/NDT.S147305 29042783 PMC5634395

[24] Chang JPC Su KP. Nutritional neuroscience as mainstream of psychiatry: the evidence-based treatment guidelines for using omega-3 fatty acids as a new treatment for psychiatric disorders in children and adolescents. *Clin Psychopharmacol Neurosci.* (2020) 18:469–83. 10.9758/cpn.2020.18.4.469 33124582 PMC7609218

[25] Lázaro CP Pondé MP. Narratives of mothers of children with autism spectrum disorders: focus on eating behavior. *Trends Psychiatry Psychother.* (2017) 39:180–7. 10.1590/2237-6089-2017-0004 28977073

[26] Sanctuary MR Kain JN Angkustsiri K German JB. Dietary considerations in autism spectrum disorders: the potential role of protein digestion and microbial putrefaction in the gut-brain axis. *Front Nutr.* (2018) 18:40. 10.3389/fnut.2018.00040 29868601 PMC5968124

[27] Cristiano C Lama A Lembo F Mollica MP Calignano A Mattace Raso G. Interplay between peripheral and central inflammation in autism spectrum disorders: possible nutritional and therapeutic strategies. *Front Physiol.* (2018) 7:184. 10.3389/fphys.2018.00184 29563885 PMC5845898

[28] Mazahery H Conlon CA Beck KL Mugridge O Kruger MC Stonehouse W Inflammation (IL-1β) modifies the effect of vitamin d and omega-3 long chain polyunsaturated fatty acids on core symptoms of autism spectrum disorder. *Proceedings.* (2020) 12:661. 10.3390/nu12030661 32121236 PMC7146497

[29] Lord C Risi S Lambrecht L Cook EH Leventhal BL Dilavore PC The autism diagnostic observation schedule-generic: a standard measure of social and communication deficits associated with the spectrum of autism. *J Autism Dev Disord.* (2000) 30:205–23. 11055457

[30] Fernández-Ballart JD Piñol JL Zazpe I Corella D Carrasco P Toledo E Relative validity of a semi-quantitative food-frequency questionnaire in an elderly Mediterranean population of Spain. *Br J Nutr.* (2010) 103:1808–16. 10.1017/S0007114509993837 20102675

[31] Aranceta-Bartrina J Partearroyo T López-Sobaler AM Ortega RM Varela-Moreiras G Serra-Majem L Updating the food-based dietary guidelines for the Spanish population: the Spanish society of community nutrition (senc) proposal. *Nutrients.* (2019) 11:2675. 10.3390/nu11112675 31694249 PMC6893611

[32] European Food Safety Authority. Scientific Opinion on nutrient requirements and dietary intakes of infants and young children in the European Union. *EFSA J.* (2013) 11:3408.

[33] Plaza-Diaz J Flores-Rojas K de la Torre-Aguilar MJ Gomez-Fernández AR Martín-Borreguero P Perez-Navero JL Dietary patterns, eating behavior, and nutrient intakes of spanish preschool children with autism spectrum disorders. *Nutrients.* (2021) 13:3551. 10.3390/nu13103551 34684552 PMC8541028

[34] Cohen IL Sudhalter V. *The PDD Behavior Inventory.* Lutz, FL: Psychol Assess Resour. (2005).

[35] Scholper E Van Bourgondien ME Wellman GJLS. *Childhood Autism Rating Scale-(CARS2).* Los Angeles, CA: West Psychol Serv (2010).

[36] Goodman R. The strengths and difficulties questionnaire: a research note. *J Child Psychol Psychiatry.* (1997) 38:581–6. 10.1111/j.1469-7610.1997.tb01545.x 9255702

[37] Lepage G Roy CC. Specific methylation of plasma nonesterified fatty acids in a one-step reaction. *J Lipid Res.* (1988) 29:227–35. 10.1016/s0022-2275(20)38553-9 3367090

[38] Gil-Campos M Larqué E Ramírez-Tortosa MC Linde J Villada I Cañete R Changes in plasma fatty acid composition after intake of a standardised breakfast in prepubertal obese children. *Br J Nutr.* (2008) 99:909–17. 10.1017/S0007114507831722 17903339

[39] Rodrigues DH Rocha NP Da Cunha Sousa LF Barbosa IG Kummer A Teixeira AL. Circulating levels of neurotrophic factors in autism spectrum disorders. *Neuroendocrinol Lett.* (2014) 35:380–4. 25275256

[40] Gomez-Fernandez A de la Torre-Aguilar MJ Gil-Campos M Flores-Rojas K Cruz-Rico MD Martin-Borreguero P Children with autism spectrum disorder with regression exhibit a different profile in plasma cytokines and adhesion molecules compared to children without such regression. *Front Pediatr.* (2018) 6:264. 10.3389/fped.2018.00264 30320048 PMC6169449

[41] Klingel SL Metherel AH Irfan M Rajna A Chabowski A Bazinet RP EPA and DHA have divergent effects on serum triglycerides and lipogenesis, but similar effects on lipoprotein lipase activity: a randomized controlled trial. *Am J Clin Nutr.* (2019) 110:1502–9. 10.1093/ajcn/nqz234 31535138

[42] Mak IL Cohen TR Vanstone CA Weiler HA. Increased adiposity in children with obesity is associated with low red blood cell omega-3 fatty acid status and inadequate polyunsaturated fatty acid dietary intake. *Pediatr Obes.* (2020) 15:e12689. 10.1111/ijpo.12689 32662950

[43] Demonty I Langlois K Greene-Finestone LS Zoka R Nguyen L. Proportions of long-chain ω-3 fatty acids in erythrocyte membranes of Canadian adults: results from the Canadian health measures survey 2012–2015. *Am J Clin Nutr.* (2021) 113:993–1008. 10.1093/ajcn/nqaa401 33675340

[44] Drover JR Hoffman DR Castañeda YS Morale SE Garfield S Wheaton DH Cognitive function in 18-month-old term infants of the DIAMOND study: a randomized, controlled clinical trial with multiple dietary levels of docosahexaenoic acid. *Early Hum Dev.* (2011) 87:223–30. 10.1016/j.earlhumdev.2010.12.047 21295417

[45] Madore C Leyrolle Q Lacabanne C Benmamar-Badel A Joffre C Nadjar A Neuroinflammation in autism: plausible role of maternal inflammation, dietary omega 3, and microbiota. *Neural Plast.* (2016) 2016:3597209. 10.1155/2016/3597209 27840741 PMC5093279

[46] Mazahery H Stonehouse W Delshad M Kruger MC Conlon CA Beck KL Relationship between long chain n-3 polyunsaturated fatty acids and autism spectrum disorder: systematic review and meta-analysis of case-control and randomised controlled trials. *Nutrients.* (2017) 9:155. 10.3390/nu9020155 28218722 PMC5331586

[47] Al-Farsi YM Waly MI Deth RC Al-Sharbati MM Al-Shafaee M Al-Farsi O Impact of nutrition on serum levels of docosahexaenoic acid among omani children with autism. *Nutrition.* (2013) 29:1142–6. 10.1016/j.nut.2013.03.009 23800562

[48] Jory J. Abnormal fatty acids in Canadian children with autism. *Nutrition.* (2016) 32:474–7. 10.1016/j.nut.2015.10.019 26746679

[49] Das UN. Autism as a disorder of deficiency of brain-derived neurotrophic factor and altered metabolism of polyunsaturated fatty acids. *Nutrition.* (2013) 29:1175–85. 10.1016/j.nut.2013.01.012 23911220

[50] van Elst K Bruining H Birtoli B Terreaux C Buitelaar JK Kas MJ. Food for thought: dietary changes in essential fatty acid ratios and the increase in autism spectrum disorders. *Neurosci Biobehav Rev.* (2014) 45:369–78. 10.1016/j.neubiorev.2014.07.004 25025657

[51] Domenichiello AF Kitson AP Bazinet RP. Is docosahexaenoic acid synthesis from α-linolenic acid sufficient to supply the adult brain? *Prog Lipid Res.* (2015) 59:54–66. 10.1016/j.plipres.2015.04.002 25920364

[52] Katan MB Deslypere JP Van Birgelen APJM Penders M Zegwaard M. Kinetics of the incorporation of dietary fatty acids into serum cholesteryl esters, erythrocyte membranes, and adipose tissue: an 18-month controlled study. *J Lipid Res.* (1997) 38:2012–22. 10.1016/s0022-2275(20)37132-7 9374124

[53] Voigt RG Mellon MW Katusic SK Weaver AL Matern D Mellon B Dietary docosahexaenoic acid supplementation in children with autism. *J Pediatr Gastroenterol Nutr.* (2014) 58:715–22. 10.1097/MPG.0000000000000260 24345834

[54] Parellada M Llorente C Calvo R Gutierrez S Lázaro L Graell M Randomized trial of omega-3 for autism spectrum disorders: effect on cell membrane composition and behavior. *Eur Neuropsychopharmacol.* (2017) 27:1319–30. 10.1016/j.euroneuro.2017.08.426 28935269

[55] Cho HP Nakamura M Clarke SD. Cloning, expression, and fatty acid regulation of the human Δ-5 desaturase. *J Biol Chem.* (1999) 274:37335–9. 10.1074/jbc.274.52.37335 10601301

[56] Cho HP Nakamura MT Clarke SD. Cloning, expression, and nutritional regulation of the mammalian Delta-6 desaturase. *J Biol Chem.* (1999) 274:471–7. 10.1074/jbc.274.1.471 9867867

[57] Boyd BA Watson LR Reszka SS Sideris J Alessandri M Baranek GT Efficacy of the ASAP intervention for preschoolers with ASD: a cluster randomized controlled trial. *J Autism Dev Disord.* (2018) 48:3144–62. 10.1007/s10803-018-3584-z 29691794

[58] Zhou B Xu Q Li H Zhang Y Wang Y Rogers SJ Effects of parent-implemented early start denver model intervention on Chinese toddlers with autism spectrum disorder: a non-randomized controlled trial. *Autism Res.* (2018) 11:654–66. 10.1002/aur.1917 29412514

[59] James S Montgomery P Williams K. Omega-3 fatty acids supplementation for autism spectrum disorders (ASD). *Cochrane Database Sys Rev.* (2011) 9:CD007992. 10.1002/14651858.CD007992.pub2 22071839 PMC13606348

[60] Yui K. [Useful pharmacologic treatment in impaired social interaction in autism spectrum disorders]. *Seishin Shinkeigaku Zasshi.* (2012) 114:934–40. 23012855

[61] Calder PC. Omega-3 polyunsaturated fatty acids and inflammatory processes: nutrition or pharmacology? *Br J Clin Pharmacol.* (2013) 75:645–62. 10.1111/j.1365-2125.2012.04374.x 22765297 PMC3575932

[62] Hu CC Xu X Xiong GL Xu Q Zhou BR Li CY Alterations in plasma cytokine levels in chinese children with autism spectrum disorder. *Autism Res.* (2018) 11:989–99. 10.1002/aur.1940 29522267

[63] Al-Ayadhi LY Mostafa GA. Elevated serum levels of macrophage-derived chemokine and thymus and activation-regulated chemokine in autistic children. *J Neuroinflammation.* (2013) 19:72. 10.1186/1742-2094-10-72 23782855 PMC3704803

[64] Sheikh AM Li X Wen G Tauqeer Z Brown WT Malik M. Cathepsin D and apoptosis related proteins are elevated in the brain of autistic subjects. *Neuroscience.* (2010) 165:363–70. 10.1016/j.neuroscience.2009.10.035 19854241

[65] Malik M Sheikh AM Wen G Spivack W Brown WT Li X. Expression of inflammatory cytokines, Bcl2 and cathepsin D are altered in lymphoblasts of autistic subjects. *Immunobiology.* (2011) 216:80–5. 10.1016/j.imbio.2010.03.001 20399529

