# Supplementary material for: Docosahexaenoic and Eicosapentaenoic Intervention Modifies Plasma and Erythrocyte Omega-3 Fatty Acid Profiles But Not the Clinical Course of Children With Autism Spectrum Disorder: A Randomized Control Trial
Source: Front Nutr. 2022 Mar 29;9:790250. doi: 10.3389/fnut.2022.790250 (PMC9002234; doi:10.3389/fnut.2022.790250)
Supplement: Supplementary file 1 [file Data_Sheet_1.pdf]

BIOSEARCH S.A.
C/Camino de Purchil, 66 - 18004 Granada (España)
C/González Dávila, 18, 6º Pl - 28031Madrid (España)
Teléf.: 91 380 29 73 Fax: 91 380 22 79
E-mail: sales@biosearchlife.com
Web: http://www.biosearchlife.com

FICHA TÉCNICA DE PRODUCTO

Producto: **EUPOLY-3® DHA Infant**

Composición: *Aceite de pescado refinado y estabilizado mediante la adición de lecitina de soja, tocoferoles de origen natural y palmitato de ascorbilo.*

Código de producto: **EUP007**

Apariencia: *Líquido ligeramente amarillento con olor y sabor característico de aceite de pescado.*

PARÁMETRO	ESPECIFICACIÓN	OBSERVACIONES
Los siguientes parámetros se analizan sobre todos los lotes de producto final		
**Índice de acidez**	*máximo* **0,8**	mgKOH/g
**Índice de peróxidos**	*máximo* **2,0**	mEq O_2/kg
**Índice de anisidina**	*máximo* **15**	
**TOTOX**	*máximo* **19**	(2xI.Peróxidos + I. Anisisdina)
**DHA – (C22:6n3)**	*mínimo* **25**	% GC área (25 picos)
**EPA + DHA**	*mínimo* **30,0**	% GC área (25 picos)
**Total Omega-3**	*mínimo* **33**	% GC área (25 picos)
**Estabilidad oxidativa**	*mínimo* **8**	Horas Rancimat (100 °C)
Los siguientes parámetros se analizan sobre la materia prima antes del refinado (punto crítico según sistema HACCP) y de forma periódica (3-5 muestras por año) sobre el producto final.		
**Pérdida por desecación (102 °C)**	*máximo* **0,3**	g/100 g
**Plomo**	*máximo* **0,1** mg/kg	ICP-MS
**Cadmio**	*máximo* **0,1** mg/kg	ICP-MS
**Mercurio**	*máximo* **0,1** mg/kg	ICP-MS
**Arsénico**	*máximo* **0,1** mg/kg	ICP-MS
**PCDD/PCDFs**	*máximo* **1,75** pg WHO-TEQ/g	HRGC-HRMS
**PCDD/PCDFs + PCBs-similares a dioxinas**	*máximo* **6,0** pg WHO-TEQ/g	HRGC-HRMS
**Suma CIEM-6**	*máximo* **200** ng/g grasa	HRGC-HRMS
**PAH4**	*máximo* **0,01** mg/kg	GC-MS (QQQ)
**Pesticidas** (OP + OC + piretroides)	Conforme a legislación en vigor	LC-MS (QQQ) y GC-MS (QQQ)
**Benzo (a) pireno**	*máximo* **2,0** ng/g	GC-MS (QQQ)

Almacenamiento: **Conservar, en el envase original sin abrir, en lugar seco protegido de la luz directa y de fuentes de calor.**

Consumo preferente: **A temperatura ambiente (preferiblemente por debajo de 25 °C), 8 meses desde la fecha de fabricación.**

Antes de su uso, atemperar el producto entre 15 y 25 °C al menos durante 12 h, y agitar vigorosamente para una correcta homogeneización del aceite.

Una vez abierto el envase, proteger de la luz y el aire y usar en los 3-4 días siguientes.

Aprobado-QA
07/11/2013

AENOR ER Empresa Registrada UNE-EN ISO 9001 ER-1255/2011
IQNet CERTIFIED MANAGEMENT SYSTEM
AENOR ER Seguridad Alimentaria UNE-EN ISO 22000 SA-0035/2011
